# The *PRKD1* promoter is a target of the KRas-NF-κB pathway in pancreatic cancer

**DOI:** 10.1038/srep33758

**Published:** 2016-09-21

**Authors:** Heike Döppler, Richard Panayiotou, Elizabeth M. Reid, Willibroad Maimo, Ligia Bastea, Peter Storz

**Affiliations:** 1 Department of Cancer Biology, Mayo Clinic Comprehensive Cancer Center, Mayo Clinic, Jacksonville, FL 32224, USA

## Abstract

Increased expression of *PRKD1* and its gene product protein kinase D1 (PKD1) are linked to oncogenic signaling in pancreatic ductal adenocarcinoma, but a direct functional relationship to oncogenic KRas has not been established so far. We here describe the *PRKD1* gene promoter as a target for oncogenic KRas signaling. We demonstrate that KRas-induced activation of the canonical NF-κB pathway is one mechanism of how *PRKD1* expression is increased and identify the binding sites for NF-κB in the *PRKD1* promoter. Altogether, these results describe a novel mechanism governing *PRKD1* gene expression in PDA and provide a functional link between oncogenic KRas, NF-κB and expression of *PRKD1*.

Activating mutations of *KRAS* are detected in over 95% of human pancreatic adenocarcinoma (PDA)^1^; and genetic mouse models have demonstrated that acquisition of oncogenic KRas is an initial event leading to pancreatic cancer^2^. Oncogenic KRas upregulates the epidermal growth factor receptor (EGF-R) and its ligands^3^, which leads to additional activation of wildtype KRas^4^,^5^; and activity of both pathways are needed for pancreatic tumorigenesis. Major downstream signaling cascades activated by active KRas in pancreatic cancer are the PI3-K/PDK1/Akt and Raf/MEK1/2/ERK1/2 pathways^6^.

In addition to oncogenic KRas mutations, high activities of both, canonical and non-canonical (alternative) nuclear factor-κB (NF-κB) activation pathways are hallmarks of human PDA^7^,^8^,^9^,^10^. Although constitutively-active in PDA and PDA cell lines, non-canonical activation of the p52/RelB NF-κB complex seems independent of oncogenic KRas^11^, and is mediated through proteasomal downregulation of TNF receptor-associated factor 2 (TRAF2) and subsequent stabilization and activation of NF-κB-inducing kinase (NIK)^9^. Activation of IKKβ and subsequent phosphorylation and downregulation of IκBα represents the canonical NF-κB signaling pathway that results in formation of a p50/p65 NF-κB complex. This pathway is constitutively-active in human PDA cells, due to oncogenic KRas^8^,^12^, and can be further increased by inflammatory cytokines^13^.

NF-κB transcription factors have been shown to regulate expression of genes involved in all aspects of tumor formation and progression. Targets regulated by increased activities of NF-κB transcription factors in PDA are survival genes such as survivin^14^ and Bcl-2^15^, or genes regulating chemoresistance such as P-gp and XIAP^14^,^15^. Overall outcome of aberrant NF-κB signaling is the amplification of KRas activity and an increase in proliferation, survival and acquisition of chemoresistance^7^,^9^,^16^. In addition to their roles in established tumors, both NF-κB pathways also contribute to the development of PDA, for example, by inducing EGF-R and its ligands TGFα and EGF^3^.

*PRKD1* and its gene product serine-threonine kinase protein kinase D1 (PKD1) are not expressed in normal pancreatic acinar cells^17^,^18^, but contribute to insulin secretion in islets^19^. PKD1 mRNA and protein expression is upregulated in acinar cells during early events that contribute to formation of pre-neoplastic lesions^18^. Consequently, it remains elevated in human patient samples of PDA^20^. However, there is no data available correlating PKD1 expression or activity with patient outcome. Overexpression or activation of PKD1 in PDA cell lines functionally was linked to increased proliferation and survival^20^,^21^,^22^,^23^,^24^,^25^,^26^,^27^. *In vivo*, an acinar cell-specific knockout of PKD1 significantly decreased the formation and progression of KRas-induced precancerous lesions^18^; and an inhibition of PKD decreased orthotopic growth of pancreatic tumor cell lines in mice^22^.

Although increased expression of PKD1 correlates with presence of oncogenic KRas in precancerous pancreatic lesions^18^, only little is known of how the *PRKD1* promoter is regulated during cancer development. By showing that KRas-mediated induction of canonical NF-κB signaling regulates the *PRKD1* promoter we here describe a novel mechanism that governs PKD1 expression in PDA.

## Results

### *Kras* regulates PKD1 expression by activating *PRKD1* gene transcription

**Figure 1:**
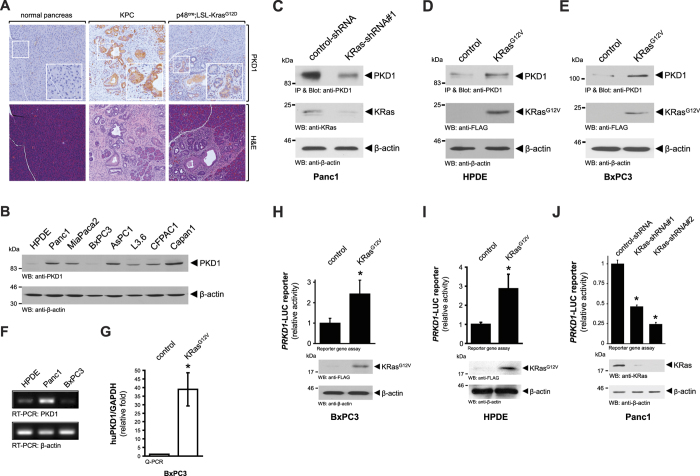
KRas regulates PKD1 expression by activating *PRKD1* gene transcription. (**A**) Sections of normal mouse pancreas, pancreata from 18 week old LSL-Kras^G12D/+^;p53^R172H/+^;Pdx1^cre/+^(KPC) mice or pancreata from 10 week old p48^cre/+^;LSL-Kras^G12D/+^(KC) mice were analyzed by H&E staining and by immunohistochemistry for PKD1 expression. Shown is a characteristic area of the pancreas. (**B**) Whole cell lysates of indicated PDA cell lines or normal control (HPDE) were analyzed by Western blotting for expression of PKD1 (anti-PKD1). Probing lysates for β-actin (anti-β-actin) served as loading control. (**C**) Panc1 cells were transfected with control-shRNA or shRNA targeting expression of KRas as indicated. 48 hours after transfection cells were lysed, PKD1 was immunoprecipitated (anti-PKD1), samples separated on SDS-PAGE, and analyzed by immunoblotting for PKD1 expression (anti-PKD1). In addition, lysates were analyzed by Western blotting for efficient knockdown (anti-KRas). Probing lysates for β-actin (anti-β-actin) served as a loading control. (**D,E**) HPDE or BxPC3 cells were transfected with KRas^G12V^ or control vector as indicated. 48 hours after transfection cells were lysed, PKD1 was immunoprecipitated (anti-PKD1 antibody), samples separated on SDS-PAGE and analyzed by immunoblotting for PKD1 expression (anti-PKD1 antibody). In addition, lysates were analyzed by Western blotting for KRas knockdown (anti-KRas) and for β-actin (anti-β-actin) as a loading control. (**F**) Indicated cell lines were cultivated under normal growth conditions. mRNA was isolated and the expression of PKD1 and β-actin was detected by RT-PCR. (**G**) BxPC3 cell were transfected with KRas^G12V^ or control vector. 48 hours after transfection a qPCR was performed. Shown is relative fold PKD1 expression normalized to GAPDH. The asterisk indicates statistical significance. (**H–J**) BxPC3 (**H**) or HPDE (**I**) cells were transfected with KRas^G12V^ or vector control, *PRKD1*-luciferase reporter and renilla-luciferase reporter; Panc1 (**J**) cells were transfected with control-shRNA or shRNA targeting KRas (two different sequences, #1 and #2), *PRKD1*-luciferase reporter and renilla-luciferase reporter. 48 hours after transfection cells were lysed, and reporter gene assays performed. In addition lysates of H, I were analyzed by Western blot for KRas^G12V^ expression (anti-FLAG), lysates of J for knockdown of KRas (anti-KRas), as well as for β-actin (anti-β-actin) as loading controls.

Expression of PKD1 was shown to be elevated in human samples of pancreatic cancer^20^, but the mechanisms of how this is achieved remain unclear. When comparing PKD1 expression in normal mouse pancreas to a genetic model in which mice develop pancreatic cancer (LSL-Kras^G12D/+^; p53^R172H/+^; Pdx1^cre/+^; KPC mouse), we detected increased levels of PKD1 in pancreatic tumors (Fig. 1A). In order to determine if this is due to either loss of p53 function, or gain of KRas activity, we included another animal model, in which oncogenic KRas is expressed under a pancreas cell specific promoter (p48^cre^;LSL-Kras^G12D^; KC mouse). The pancreata of these mice usually show Kras^G12D^-caused regions of acinar cell metaplasia and PanIN1A/B and PanIN2 lesions. The presence of oncogenic KRas alone resulted in increased PKD1 expression in abnormal pancreas structures (Fig. 1A).

In order to determine if mutant KRas can lead to PKD1 expression, we first analyzed a panel of PDA cell lines as well as normal human pancreatic ductal epithelial (HPDE) cells as a control. We found PKD1 protein highly expressed in all PDA cell lines except BxPC3 and HPDE control cells (Fig. 1B). Interestingly, HPDE and BxPC3 express wildtype KRas, whereas all other PDA cells lines with elevated PKD1 levels express oncogenic versions of KRas. To test if presence of mutant KRas and expression of PKD1 are directly linked, we knocked-down oncogenic KRas in Panc1 cells and found that this correlated with decreased levels of PKD1 expression (Fig. 1C). In addition, expression of a mutant KRas in HPDE or BxPC3 cells led to an increase in PKD1 protein expression (Fig. 1D,E). Comparison of mRNA expression of Panc1 to HPDE and BxPC3 using RT-PCR analyses indicated that changes in protein levels may be linked to mRNA expression (Fig. 1F). Moreover, ectopic expression of KRas^G12V^ in BxPC3 cells led to a dramatic increase in expression PKD1 mRNA (Fig. 1G). This prompted us to determine if altered PKD1 expression by mutant KRas is regulated at the *PRKD1* promoter level. Therefore, we introduced an oncogenic variant of KRas together with a *PRKD1*-luciferase reporter into BxPC3 (Fig. 1H) or HPDE (Fig. 1I) cells, and performed reporter gene assays to determine promoter activity. In both cell lines the expression of mutant KRas induced the expression of the *PRKD1* promoter. A reverse genetics approach in which we knocked-down KRas in Panc1 cells confirmed the link between presence of mutant KRas and increased *PRKD1* promoter activity (Fig. 1J).

### Oncogenic KRas activates the canonical NF-κB pathway to induce *PRKD1* expression

**Figure 2:**
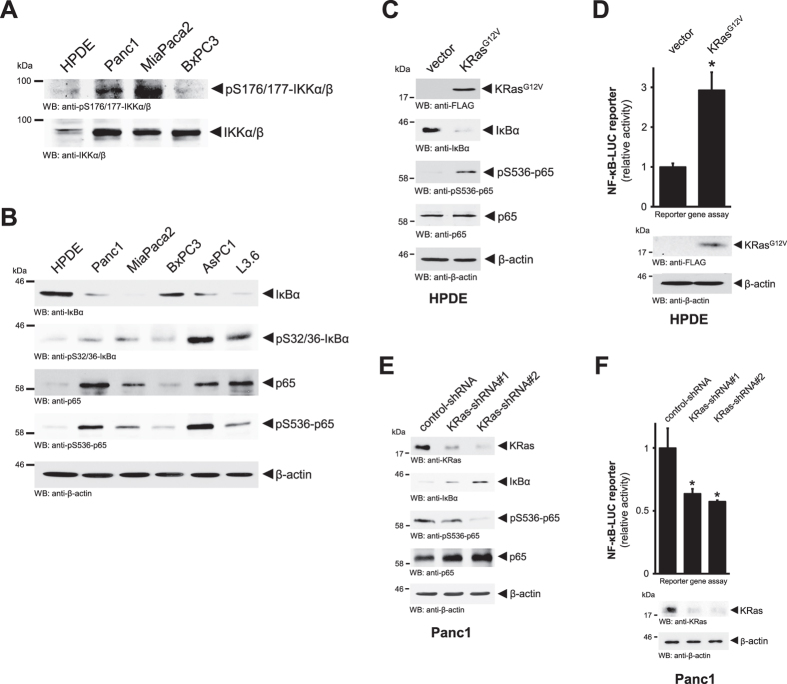
Oncogenic Kras activates the canonical NF-κB pathway. (**A**) Whole cell lysates of indicated PDA cell lines or normal control (HPDE) were analyzed by Western blotting for an activating phosphorylation of IKKα/β (anti-pS176/177-IKKα/β) or total protein (anti-IKKα/β). (**B**) Whole cell lysates of indicated PDA cell lines or normal control (HPDE) were analyzed by Western blotting for presence of IκBα phosphorylation (anti-pS32/36-IκBα), total IκBα, phosphorylation of p65 (anti-pS536-p65) or total p65. Probing lysates for β-actin (anti-β-actin) served as loading control. (**C**) HPDE cells were transfected with KRas^G12V^ or control vector as indicated. 24 hours after transfection lysates were analyzed by Western blotting for expression of KRas^G12V^ (anti-FLAG) or downregulation of IκBα (anti-IκBα), phosphorylation of p65 (anti-pS536-p65) or total p65. Probing lysates for β-actin (anti-β-actin) served as loading control. (**D**) HPDE cells were transfected with KRas^G12V^ or control vector, NF-κB-luciferase reporter and renilla-luciferase reporter, as indicated. 24 hours after transfection cells were lysed, and reporter gene assays performed. In addition lysates were analyzed by Western blot for KRas^G12V^ expression (anti-FLAG) as well as for β-actin (anti-β-actin). (**E**) Panc1 cells were transfected with control-shRNA or shRNA targeting KRas (two different sequences, #1 and #2), as indicated. 24 hours after transfection lysates were analyzed by Western blotting for expression of endogenous KRas (anti-KRas) or presence of IκBα (anti-IκBα), phosphorylation of p65 (anti-pS536-p65) or total p65. Probing lysates for β-actin (anti-β-actin) served as loading control. (**F**) Panc1 cells were transfected with control-shRNA or shRNA targeting KRas (two different sequences, #1 and #2), as indicated. 24 hours after transfection cells were lysed, and reporter gene assays performed. In addition lysates were analyzed by Western blot for knockdown of KRas (anti-KRas), as well as for β-actin (anti-β-actin).

The canonical NF-κB pathway has been implicated as one of the main targets for oncogenic KRas in PDA^12^,^28^. In the four cell lines primarily used in our study, we found a correlation between expression of oncogenic versions of KRas (Panc1 and MiaPaca2) and basal activity of IKKα/β complex (Fig. 2A). We then analyzed a larger panel of cell lines for *bona fide* markers of activation of the canonical pathway such as phosphorylation and downregulation of IκBα and phosphorylation of p65 at S536 (Fig. 2B). We found that presence of oncogenic versions of KRas correlates with a decrease of expression of IκBα and an increase in protein levels and S536 phosphorylation of p65 (Fig. 2B). Cells such as HPDE and BxPC3 that express wildtype KRas show reverse occurrence of these components of the canonical pathway (Fig. 2B). To establish a direct functional relationship between oncogenic KRas and canonical NF-κB signaling, we expressed KRas^G12V^ in HPDE and found that this led to downregulation of IκBα and increased phosphorylation of p65 (Fig. 2C). NF-κB reporter gene assays demonstrated that this indeed leads to activation of NF-κB (Fig. 2D). Similarly, the knockdown of endogenous KRas in Panc1 cells increased presence of IκBα, decreased phosphorylation of p65 (Fig. 2E) and decreased the basal activity of NF-κB (Fig. 2F).

**Figure 3:**
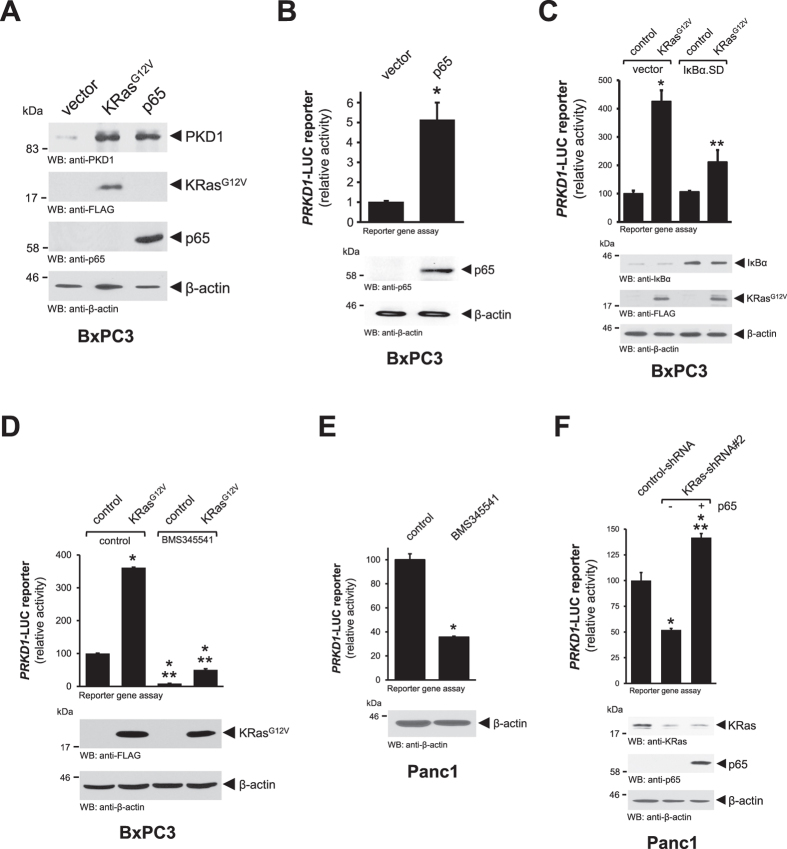
Oncogenic Kras induces *PRKD1* expression via the canonical NF-κB pathway. (**A**) BxPC3 cells were transfected with vector control, KRas^G12V^ or p65 as indicated. 24 hours after transfection lysates were analyzed by Western blotting for expression of endogenous PKD1 (anti-PKD1). Probing lysates for KRas^G12D^ (anti-FLAG), p65 (anti-p65) or β-actin (anti-β-actin) served as expression or loading controls. (**B**) BxPC3 cells were transfected with vector control or p65 and *PRKD1*-luciferase and renilla-luciferase reporters. 24 hours after transfection cells were lysed, and reporter gene assays performed. In addition, probing lysates for p65 (anti-p65) or β-actin (anti-β-actin) served as expression or loading controls. (**C**) BxPC3 cells were co-transfected with vector control, IκBα.SD or KRas^G12V^ and *PRKD1*-luciferase and renilla-luciferase reporters. 24 hours after transfection cells were lysed, and reporter gene assays performed. Probing lysates for IκBα (anti-IκBα), KRas^G12V^ (anti-FLAG) or β-actin (anti-β-actin) served as expression or loading controls. (**D**) BxPC3 cells were co-transfected with vector control or KRas^G12V^ and *PRKD1*-luciferase and renilla-luciferase reporters and, after 5 hours, treated with BMS345541 (10 μM). 24 hours after stimulation cells were lysed, and reporter gene assays performed. Probing lysates for KRas^G12V^ (anti-FLAG) or β-actin (anti-β-actin) served as expression or loading controls. (**E**) Panc1 cells were co-transfected with *PRKD1*-luciferase and renilla-luciferase reporters and then treated with BMS345541 (10 μM). 24 hours after stimulation cells were lysed, and reporter gene assays performed. Probing lysates for β-actin (anti-β-actin) served as internal control. (**F**) Panc1 cells were transfected with control-shRNA or shRNA targeting KRas, as well as vector control or p65 and *PRKD1*-luciferase and renilla-luciferase reporters, as indicated. 24 hours after transfection cells were lysed, and reporter gene assays performed. Probing lysates for KRas (anti-KRas), p65 (anti-p65) or β-actin (anti-β-actin) served as expression or loading controls.

Once we established that oncogenic KRas activates the canonical NF-κB pathway we investigated if this pathway is involved in KRas-mediated regulation of *PRKD1* gene expression. Similar to oncogenic KRas, the ectopic expression of p65 in BxPC3 cells increased protein levels of PKD1 (Fig. 3A). This is due to increased activation of the *PRKD1* promoter as determined in luciferase reporter assays in which cells were transfected with p65 (Fig. 3B). In order to determine if NF-κB acts downstream of oncogenic KRas to induce *PRKD1*, we blocked downstream signaling by expressing a “super-dominant” allele of IκBα (IκBα.SD) that cannot be phosphorylated and therefore blocks induction of NF-κB. Both BxPC3 and HPDE cells when expressing IκBα.SD showed a significant decrease in KRas^G12V^-induced activation of the *PRKD1* promoter, indicating that NF-κB indeed operates downstream of oncogenic KRas (Fig. 3C and Supplemental Fig. S1A). Similarly, chemical inhibition of IKKα/β using BMS345541, blocked KRas^G12V^-mediated induction of the *PRKD1* promoter in BxPC3 cells (Fig. 3D), and also decreased basal promoter activity in Panc1 cells (Fig. 3E). Eventually, we also show that ectopic expression of p65 in Panc1 cells can rescue *PRKD1* expression in presence of shRNA that targets endogenous KRas after 24 or 48 hours (Fig. 3F; Supplemental Fig. S1B). Taken together this establishes canonical NF-κB signaling downstream of oncogenic KRas as a major pathway to induce expression of *PRKD1*.

### Identification of a KRas^G12V^-regulated NF-κB binding motif in the *PRKD1* promoter

**Figure 4:**
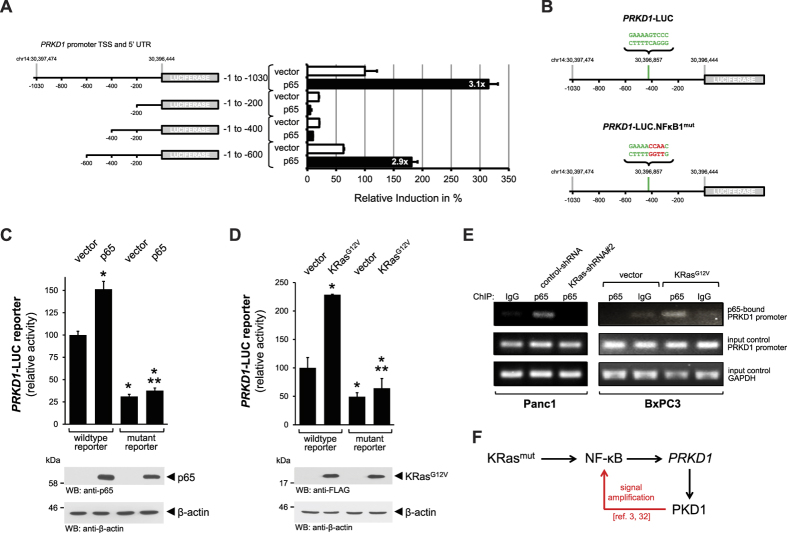
Mapping of the NF-κB site relevant for KRas^G12V^-mediated *PRKD1* promoter activation. (**A**) Cells were transfected with vector control or p65 as well as full-length or indicated truncated versions of the *PRKD1* promoter luciferase reporter and renilla-luciferase reporter, as indicated. 24 hours after transfection cells were lysed, and reporter gene assays performed. (**B**) Schematic of a NF-κB1 motif (green) in the mapped region of the *PRKD1* promoter and mutational alterations performed to destruct the motif (red). (**C**,**D**) Comparison of response of wildtype and mutant *PRKD1* promoter luciferase reporter to p65 or Kras^G12V^. Cells were transfected with vector control or p65 (**C**) or vector control and Kras^G12V^, as well as full-length wildtype or mutant versions of the *PRKD1* promoter luciferase reporter and renilla-luciferase reporter, as indicated. 24 hours after transfection cells were lysed, and reporter gene assays performed. In addition lysates were analyzed by Western blot for expression of p65 (anti-p65) or KRas^G12V^ (anti-FLAG), as well as for β-actin (anti-β-actin). (**E**) Panc1 cells were transfected with control-shRNA or shRNA targeting expression of KRas, as indicated, for 48 hours. BxPC3 cells were transfected with vector control or KRas^G12V^ as indicated. Chromatin immunoprecipitation (ChIP) was performed using anti-p65 or IgG control and ChIP of p65-bound *PRKD1* promoter was detected by PCR. Input controls show PCR for *PRKD1* promoter and GAPDH using sheared DNA as a template. (**F**) Schematic of how oncogenic KRas induces the expression of PKD1 via activation of NF-κB. Red arrows take in consideration published data showing that PKD1 also can activate NF-κB downstream of mutant KRas, this indicating a potential positive feedback loop for signal amplification.

Next we used our *PRKD1* promoter luciferase reporter construct^29^, which includes TSS and 5′UTR of the *PRKD1* gene within a 1030 nucleotide sequence comprising chr14:30,397,474 to chr14:30,396,444, and generated truncated versions thereof (Fig. 4A, schematic left side). First we used these reporter gene constructs to determine the region within *PRKD1* that responds to expression of p65. We found that expression of p65 led to a 3.1 fold induction of the full-length reporter as well as a 2.9 fold induction of the 600 nucleotide truncated version (Fig. 4A, right side; control blots shown in Supplemental Fig. S2A). Further truncations, however, blunted the induction of the reporter, indicating that NF-κB binding occurs within a stretch of 200 nucleotides (region −400 to −600; Supplemental Fig. S2B).

Using a series of prediction programs such as TESS (http://www.hsls.pitt.edu/obrc/index.php?page=URL1101824594), AliBaba2.1 (http://www.gene-regulation.com/pub/programs/alibaba2/index.html) and the SABiosciences ChIP search tool (http://www.sabiosciences.com/chipqpcrsearch.php?app=TFBS&qs=1394823976) we explored potential NF-κB binding sites in this promoter region of *PRKD1*. As a result we identified GAAAAGTCCC (chr14:30396847-chr14:30396856) as a likely NF-κB binding site (Supplemental Fig. S2B). This site shows a reverse orientation and only slightly varies to the classical GGGRNNYYCC motif^30^. Similar slight alterations to the consensus motif have been described for other NF-κB target genes such as TNFα and others^31^.

Next, we generated a *PRKD1* promoter luciferase reporter construct in which the NF-κB binding motif was destroyed by mutation to GAAAACCAAC (Fig. 4B). Comparison of this mutant to the wildtype *PRKD1* promoter luciferase reporter indeed showed that it is less responsive to expression of p65 and oncogenic KRas. For example, while the wildtype reporter shows basal activity which is further increased by ectopic expression of p65 (Fig. 4C) or KRas^G12V^ (Fig. 4D), the mutant shows decreased basal activity and cannot be further activated by expression of p65 or KRas^G12V^. Eventually we performed chromatin immunoprecipitation (ChIP) assays to show that p65/NF-κB can bind the *PRKD1* promoter in response to oncogenic KRas^G12V^. While in Panc1 cells under normal growth conditions p65 in bound to the *PRKD1* promoter, this is blocked after depletion of cells from KRas (Fig. 4E, left side). However, in BxPC3 cells under control conditions the relevant *PRKD1* promoter region was not detected in p65 immunoprecipitates, in presence of oncogenic Kras it was detected in p65 immunoprecipitates (Fig. 4E, right side). Overall our data suggest that one pathway of how *PRKD1* expression downstream of oncogenic KRas is regulated is through NF-κB (Fig. 4F).

### Correlation of presence of p65 phosphorylation and PKD1 expression in human PDA

**Figure 5:**
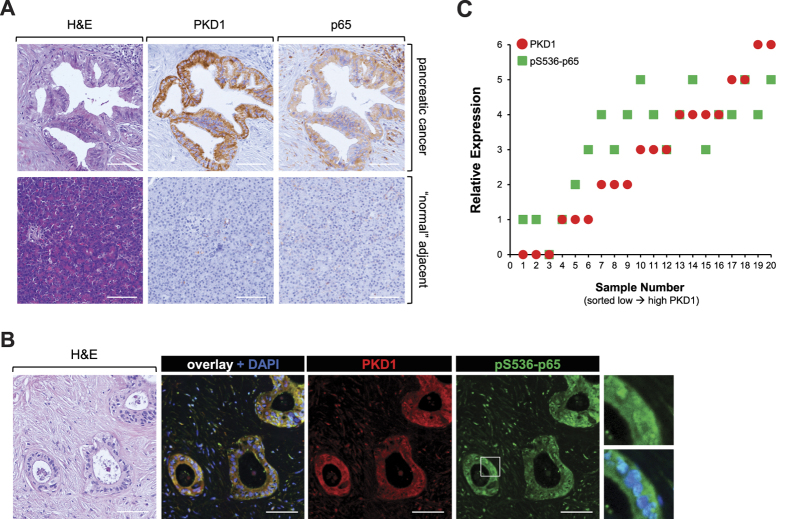
Correlation of p65 and PKD1 expression in human PDA. (**A**) Serial sections that show a representative of 20 different patient tissue samples that were stained by H&E or IHC for expression of PKD1 or p65. Shown is a representative lesion area. The bar indicates 100 μm. (**B**) Shows a representative of 20 different patient tissue samples that were co-stained by IHC-IF for PKD1 (red) or phospho-S536-p65 (green). Shown is a representative lesion area. The bar indicates 100 μm. (**C**) Relative expression of PKD1 and pS536-p65 in n = 20 patient samples. The quantitation analysis is described in Materials & Methods.

Analyses of human samples of PDA indicated strong correlation between PKD1 expression and presence of p65 in tumors, but no expression of both proteins in “normal” acinar tissue, adjacent to the tumor (Fig. 5A). We then performed a co-staining of PKD1 and pS536-p65 to determine if the presence of PKD1 correlates with active p65. Shown in Fig. 5B is an IHC-IF of a typical section of human PDA. PKD1 as well as pS536-p65 can be detected; and pS536-p65 shows a partial nuclear localization (see enhancement of inset, Fig. 5B right side). A quantitative analysis of 20 different patient samples showed a strong correlation between levels of PKD1 expression and S536-phosphorylation of p65 (Fig. 5C), further emphasizing a functional relationship between PKD1 expression and canonical NF-κB signaling in human PDA.

## Discussion

Acquisition of an oncogenic KRas is a driver mutation for pancreatic cancer, but its downstream signaling has not been fully characterized. While PKD1 expression is upregulated in pancreatic cancer cells and patient tissue^20^, gene amplifications or activating mutations have not been detected. We previously have shown that during the development of precancerous lesions PKD1 converges downstream signaling by both oncogenic KRas and wildtype KRas and that a knockout of PKD1 significantly decreases the formation of abnormal pancreatic structures^18^. In these processes that led to PanIN formation and progression to PDA, KRas upregulates PKD1 expression and activity. While PKD1 activity downstream of KRas seems to be increased via accumulation of mitochondrial reactive oxygen species (mROS)^3^, the mechanism of how KRas increases PKD1 expression levels so far was unidentified. *PRKD1* gene expression can be regulated epigenetically by DNA methylation^29^,^32^, but no transcriptional regulation has been demonstrated. Here we show that oncogenic KRas upregulates the expression of PKD1 at the promoter level through activation of the transcription factor NF-κB (Figs 1, 2, 3, 4).

High levels of canonical and alternative NF-κB signaling are hallmarks of PDA and have been linked to increased survival, proliferation and chemoresistance^7^,^9^,^33^. Oncogenic KRas can induce canonical NF-κB signaling (refs 12 and 18, Fig. 2), whereas constitutive alternative NF-κB signaling mainly seems to be due to TRAF2 downregulation leading to increased stability of NIK^9^. Our data now provide a functional link between KRas, canonical NF-κB signaling and induction of PKD1 expression. This is an interesting finding because PKD1 downstream of KRas/mROS also can activate canonical NF-κB signaling^3^. Therefore, with upregulating PKD1 expression and activity, KRas may initiate an amplification process for NF-κB signaling in PDA cells (see scheme in Fig. 4F). All together this feedback signaling loop may explain the highly-increased levels of NF-κB in PDA patients. It also may contribute to further increase KRas oncogenic functions, since PKD1 also activates IKK2^34^, which has been shown to be involved in amplifying KRas activity to pathological levels^16^. Other similar feedforward loops have been described to sustain NF-κB activity. For example, oncogenic KRas can induce IL-1α, which then activates NF-κB and its target genes IL-1α and p62. Resulting IL-1α/p62 signaling further induces and sustains NF-κB activity^12^. At this point it is unclear if there is an interface between this signaling pathway and the here described KRas-NF-κB-PKD1-NF-κB signal amplification pathway.

Another interesting aspect is that PKD1 has been shown to induce Notch signaling downstream of mutant KRas in precancerous lesions^18^. A crosstalk between Notch and canonical NF-κB signaling pathways is needed for progression of pancreatic cancer^13^, and PKD1 is a key enzyme linking KRas to Notch and NF-κB. Therefore, PKD1 could be a promising new target to prevent precancerous lesions and tumor formation, but also progression of tumors.

## Materials and Methods

### Cell Lines, Reagents and Antibodies

Human pancreas ductal epithelial (HPDE) cells were obtained from M.S. Tsao (Ontario Cancer Institute, Ontario, Canada) and maintained as previously described^9^. All other cell lines were from the American Type Culture Collection (ATCC, Manassas, VA) and were maintained as suggested by the ATCC. Lipofectamine 2000 from Invitrogen (Carlsbad, CA) or TransIT-2020 from Mirus Bio LLC (Madison, WI) was used for transfection of BxPC3, Panc1, MiaPaca2 and HPDE cells. BMS345541 was from EMD Millipore (Billerica, MA). The anti-phospho-S536-p65 (ab86299) antibody was from Abcam (Cambridge, MA), the anti-p65, anti-phospho-S32/36-IκBα (5A5), anti-IκBα and anti-phospho-S176/177-IKKα/β (2078) antibodies were from Cell Signaling Technology (Danvers, MA), the anti-p65 for ChIP, anti-IKKα/β and anti-KRas antibodies from Santa Cruz (Santa Cruz, CA), anti-FLAG (F7425) and anti-β-actin antibodies from Sigma-Aldrich (St Louis, MO). Rabbit polyclonal and mouse monoclonal PKD1-specific antibodies (used for Western blotting and immunoprecipitation) were described before^29^,^35^. The anti-PKD1 antibodies for IHC and IHC-IF were from Antibodies-online Inc. (Atlanta, GA) and from Everest Biotech (Oxfordshire, UK). Secondary HRP-linked anti-mouse or anti-rabbit antibodies were from Roche (Indianapolis, IN). Secondary Alexa Fluor 647 and Alexa Fluor 488 antibodies were from Invitrogen.

### DNA and Viral Constructs

The *PRKD1* promoter sequence was described previously^29^. A 5′-GCGAGATCTCGGCGACTTACCTTCTGGTCGACA-3′ primer was combined with 5′-CGCACGCGTGCACTGGTCCCAGGGTCCGGGCCC-3′ (200 bp fragment), 5′-CGCACGCGTAAAGTTTTTATTTTCCGTCTGGGC-3′ (400 bp fragment), 5′-CGCACGCGTCCTTCCGAGCGCGCCAGGCGGCAG-3′ (600 bp fragment), 5′-CGCACGCGTCTCAGGCCCCGGGAACTGCGGGCG-3′ (800 bp fragment), or 5′-CGCACGCGTGTAGAAGTGCGTAGTCCTCCAGCA-3′ (1030 bp fragment) primers to amplify fragments of the *PRKD1* promoter which were cloned into pGL3 via Mlu I and Bgl II sites to generate different *PRKD1*-luciferase reporter constructs. The following primers were used for destruction of the NF-κB binding site in the *PRKD1* promoter: 5′-GCCCTCCCCCAGCCCAGTTGGTTTTCCGGAAAGTTTTTAT-3′ and 5′-ATAAAAACTTTCCGGAAAACCAACTGGGCTGGGGGAGGGC-3′. The NF-κB-luciferase and renilla luciferase reporter constructs were described previously^34^. The lentiviral shRNA expression system to knock-down human KRas is commercially available from Sigma (SHDNA MISSION^®^ shRNA Plasmid DNA; St. Louis, MO, USA). Sequences used were human KRas NM_033360.2-269s1c1 (labeled: KRas-shRNA#1) and human KRas NM_004985.3-641s1c1 (labeled: KRas-shRNA#2). The expression constructs for FLAG-tagged KRas^G12V^, p65 and IκBα.SD have been described previously^18^,^34^,^36^.

### Immunoblotting, Immunoprecipitation and PAGE

To generate cell lysates, cells were washed twice with 4 °C PBS (140 mM NaCl, 2.7 mM KCl, 8 mM Na_2_HPO_4_, 1.5 mM KH_2_PO_4_, pH 7.2), lysed with lysis buffer (50 mM Tris-HCl pH7.4, 1% Triton X-100, 150 mM NaCl, 5 mM EDTA pH 7.4) plus Protease Inhibitor Cocktail (PIC, Sigma-Aldrich), vortexed, incubated on ice for 30 min and centrifuged (13,000 rpm, 15 min, 4 °C). Protein concentration was determined, lysates normalized and proteins of interest were immunoprecipitated by 1 hr incubation with 2 μg of a specific antibody (as indicated), followed by 30 min incubation with protein G-Sepharose (GE Healthcare Life Sciences, Marlborough, MA). Samples were washed 3 times with TBS (50 mM Tris-HCl pH 7.4, 150 mM NaCl) and resolved in 20 μl TBS plus 20 μl 2x Laemmli buffer. After SDS-PAGE, samples were transferred to nitrocellulose membranes and proteins of interest visualized by immunostaining.

### RT-PCR

Total RNA was isolated from cells using RNA-Bee (TEL-TEST, Friendswood,TX) according to the manufacturer’s instructions. 1 μg of RNA was reverse transcribed to cDNA using ImProm-II Reverse Transcription System from Promega. 100 ng cDNA were used as template for the PCR reaction. The primers used for PKD1 were 5′-TTCTCCCACCTCAGGTCATC-3′ and 5′-TGCCAGAGCACATAACGAAG-3′; and for β-actin 5′-CCTCGCCTTTGCCGATCC-3′ and 5′-GGATCTTCATGAGGTAGTCAGTC-3′. The reaction conditions for the PCR were: 30 seconds denaturation at 95 °C, 1 minute annealing at 60 °C and 1 minute extension at 72 °C; for 30 cycles. Resulting PCR mixtures were resolved on a 2% agarose gel.

### Quantitative Real-time PCR

Total RNA was isolated using the RNeasy kit (Qiagen, Frederick, MD) and 500 ng total RNA each sample was converted to cDNA using the High Capacity cDNA Reverse Transcriptase kit (Applied Biosystems, Bedford, MA). Quantitative real-time PCR was performed with a 7900HT Fast real time thermocycler (Applied Biosystems) and the TaqMan Universal PCR Master Mix (Applied Biosystems). The reaction included 10 ng cDNA as template and primer sets from Applied Biosystems (Hs00177037_m1 and Hs02758991_g1). Conditions: 95 °C for 20 seconds; 40 cycles of 95 °C for 1 second and 60 °C for 20 seconds. Data were collected by a Prism 7900 sequence detector and analyzed with Sequence Detection System software (Applied Biosystems). Data were normalized to human GAPDH, and mRNA levels of PKD1 were calculated using the ΔΔ*C*_T_ method and plotted as relative fold to the respective control.

### Reporter Gene Assays

As indicated, cells were transfected with 3 μg *PRKD1*-luciferase reporter (*PRKD1*-LUC), 3 μg NF-κB-luciferase reporter (NF-κB-LUC), 0.1 μg renilla luciferase reporter and 1 μg of the cDNA or shRNA of interest per well of a 6 well plate. At indicated times after transfection, cells were washed twice with ice-cold PBS, scraped in 250 μl Passive Lysis Buffer (Promega) and centrifuged (13,000 rpm, 10 min, 4 °C). Assays for luciferase activity were performed according to the Promega Luciferase assay protocol and measured using a Veritas luminometer (Symantec, Cupertino, CA). Luciferase activity of the *PRKD1*-LUC or NF-κB-LUC reporter constructs was normalized to renilla luciferase activity. Expression or knockdown of proteins was controlled by Western blot analysis, as indicated.

### Chromatin Immunoprecipitation (ChIP) Assay

ChIP assays were performed using the EZ-ChIP™ Chromatin Immunoprecipitation (ChIP) KIT from Millipore (Bedford, MA) according to the manufacturer’s protocol. 4 μg primary anti-p65 antibody or IgG control (Jackson ImmunoResearch Laboratories, West Grove, PA) were used for immunoprecipitations. Immunoprecipitates were analyzed by PCR using the following primer set: 5′-TTTCCCTCCTCCCCATCT-3′ and 5′-GAAAGTTTTGCAGCCGCT-3′ to amplify a 154 bp fragment of the human *PRKD1* promoter that includes the identified NF-κB site.

### Animal Samples

Conditional LSL-Kras^G12D/+^ mice were obtained from the NCI Mouse Repository (MMHCC) and crossed with p48^cre/+^ mice (described in ref. 18) to generate bi-transgenic p48^cre/+^;LSL-Kras^G12D/+^ mice. Pdx^cre/+^ mice and p53^R172H/+^(both described in ref. 37) and LSL-Kras^G12D^ mice were crossed to obtain KPC mice. All animal experiments were approved by the Mayo Clinic Institutional Animal Care and Use Committee (IACUC). In addition, all experiments were performed in accordance with relevant guidelines and regulations.

### Human Pancreatic Tissue Samples

De-identified patient tissues were obtained from archival materials (for which written informed consent for the use of these tissues in research was obtained from all participants) in accordance with institutional guidelines and prior Mayo Clinic Institutional Review Board (IRB) approval. All experiments were performed in accordance with relevant guidelines and regulations. Tissue samples were scored independently by two different experienced scientists. Uniform pre-established criteria were used. Immunoreactivity was graded semiquantitatively by considering the intensity of the staining in tumors. A histological score was obtained from each sample, which ranged from 0 (no immunoreaction) to 6 (maximum immunoreactivity). Reproducibility of the scoring method between observers was greater than 90%. In the remaining cases, in which discrepancies had been noted, differences were settled by consensus review of corresponding tissues.

### Immunohistochemistry

Slides were deparaffinized (1 hour, at 60 °C), dewaxed in xylene (five times, each 4 minutes) and gradually rehydrated with enthanol (100%, 95%, 75%, twice each, for 3 minutes). Then tissue samples were rinsed in water, subjected to antigen retrieval (citrate buffer pH 6.0), treated with 3% hydrogen peroxide (5 minutes) and washed with PBS containing 0.5% Tween 20. PKD1 expression was detected using anti-PKD1 antibody (for mouse samples: Antibodies-online, 1:100 in PBS/Tween; for human samples: Everest Biotech, 1:2000 in PBS/Tween); p65 was detected using anti-p65 antibody (1:1000 in PBS/Tween) and visualized using the Envision Plus Dual Labeled Polymer Kit (DAKO, Caprinteria, CA). H&E staining was performed as previously described^9^. Images were captured using ScanScope XT scanner and ImageScope software (Aperio, Vista, CA).

### Immunofluorescence Analyses of Tissues

For immunofluorescence analysis, tissue sections underwent antigen retrieval with citrate buffer at pH 6.0 for 25 minutes at 100 °C, were incubated in 3% hydrogen peroxide for 15 minutes at room temperature (RT), rinsed in PBS and blocked in serum-free Protein Block (Dako X0909) for one hour at RT. Samples were then incubated with primary antibodies (PKD1, Everest Biotech, 1:2000; and anti-pS536-p65, 1:50) diluted in Antibody Diluent (Dako S3022) overnight at 4 °C. Slides were washed three times with 0.025% Tween-20/PBS, followed by a 2 hour incubation of Alexa Fluor 488- or 647-conjugated secondary antibodies at 1:500 (Invitrogen) and DAPI (1:1000) at RT. Stained slides were washed three times with 0.025% Tween-20/PBS and mounted in Aqua-Poly/Mount (Polysciences, Inc., Warrington, PA). The fluorescent images were collected using ScanScope FL and ImageScope software (Aperio).

### Statistical Analysis

Data are presented as mean ± SD. P values were acquired with the student’s *t*-test using Graph Pad software, and p < 0.05 was considered statistically significant.

## Additional Information

**How to cite this article**: Döppler, H. *et al.* The *PRKD1* promoter is a target of the KRas-NF-κB pathway in pancreatic cancer. *Sci. Rep.* **6**, 33758; doi: 10.1038/srep33758 (2016).

## Supplementary Material

Supplementary Information
