# Supplementary material for: The PRKD1 promoter is a target of the KRas-NF-κB pathway in pancreatic cancer
Source: Sci Rep. 2016 Sep 21;6:33758. doi: 10.1038/srep33758 (PMC5030668; doi:10.1038/srep33758)
Supplement: Supplementary Information [file srep33758-s1.pdf]

Supplementary Information
for

The *PRKD1* promoter is a target of the KRas-NF-κB pathway in pancreatic cancer

Heike Döppler[1], Richard Panayiotou[1], Elizabeth M. Reid[1], Willibroad Maimo[1], Ligia Bastea[1] and Peter Storz[1,*]

[1] Department of Cancer Biology, Mayo Clinic Comprehensive Cancer Center, Mayo Clinic, Jacksonville, FL 32224, USA

* Corresponding author: Peter Storz, Mayo Clinic, Griffin Building, Room 306, 4500 San Pablo Road, Jacksonville, FL 32224. Tel: 904 953-6909, Fax: 904 953-0277, e-mail: storz.peter@mayo.edu

Figure S1, related to Figure 3

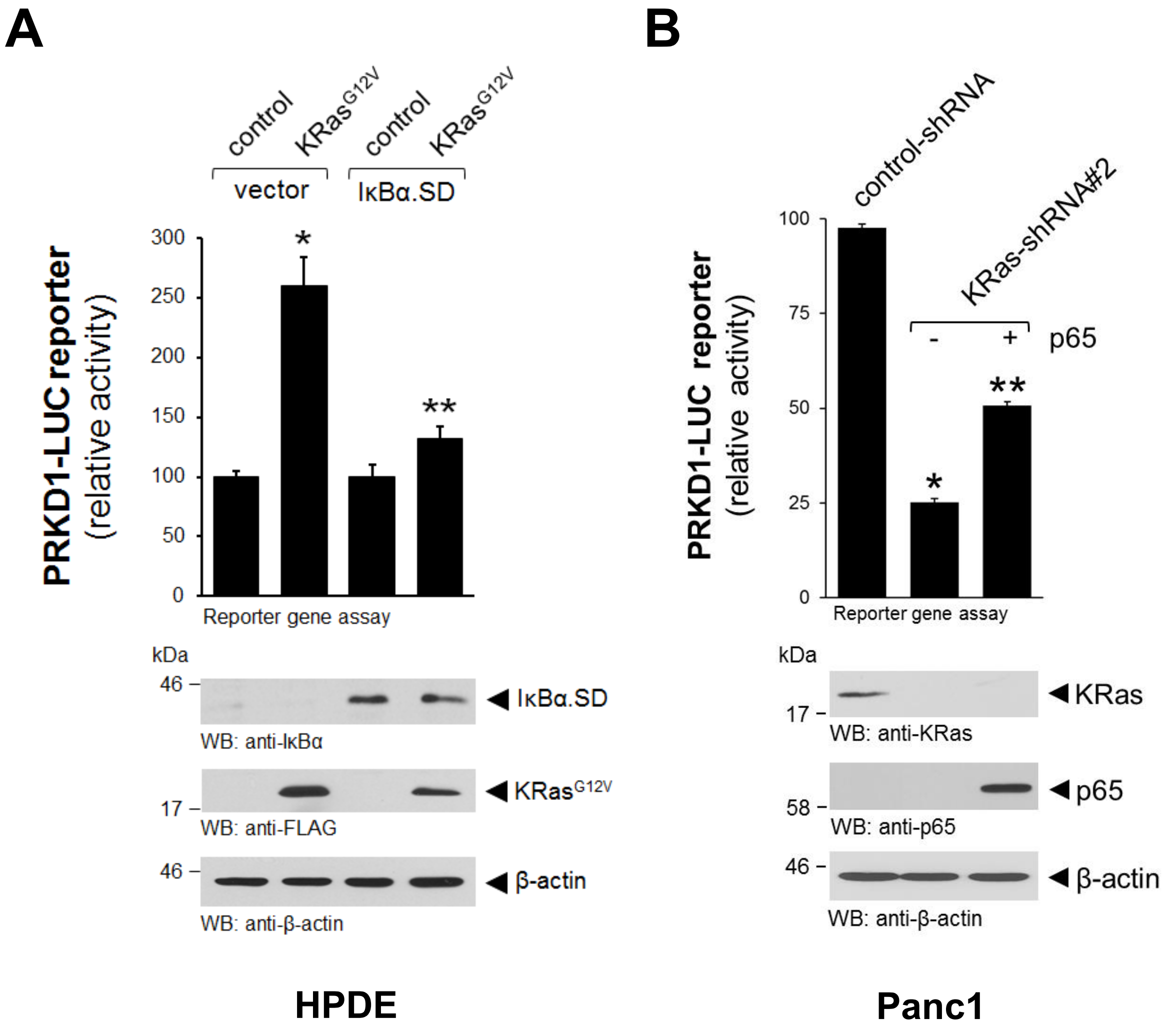

Figure S1, relates to Figure 3: **Oncogenic Kras induces *PRKD1* expression via the canonical NF-κB pathway. A:** HPDE cells were co-transfected with vector control, IκBα.SD or KRas^{G12V} and *PRKD1*-luciferase and renilla-luciferase reporters. 24 hours after transfection cells were lysed, and reporter gene assays performed. Probing lysates for IκBα (anti-IκBα), KRas^{G12V} (anti-FLAG) or β-actin (anti-β-actin) served as expression or loading controls. **B:** Panc1 cells were transfected with control-shRNA or shRNA targeting KRas, as well as vector control or p65 and *PRKD1*-luciferase and renilla-luciferase reporters, as indicated. 48 hours after transfection cells were lysed, and reporter gene assays performed. Probing lysates for KRas (anti-KRas), p65 (anti-p65) or β-actin (anti-β-actin) served as expression or loading controls.

Figure S2, related to Figure 4

A

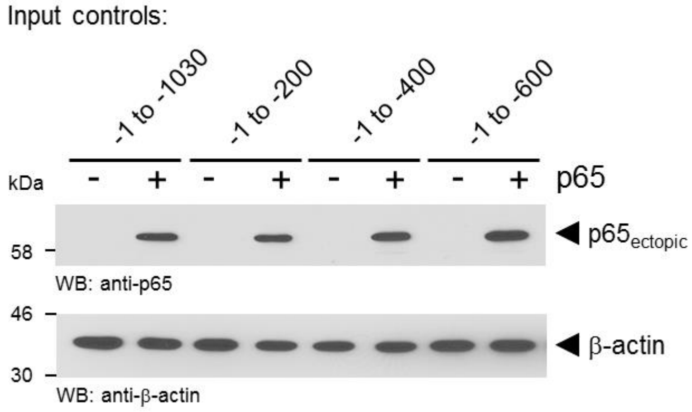

B

```
CGGCGACTTACCTTCTGGTCGACAATGGAGCAAGCCATCTCGCGGACGTGCGCCAGGCTGTAGT
CCCCGGACGAGTCCTGCAGCAGCAGCACCGGCTCACGGCTCAGGCCGATCTGCAGATGGAACGA
GATGCCCCCGACCGGGGCCGCGACAGGAGCCAAGAACGGCGCGGGCCCGGGCCCGGACCCTGGG
ACCAGTGCGGCGGCCGCTGCGGCAGCTGCCGCCGCCACGGGCAGCAGCGGACTGGGCGGCCGCA
GGACCGGAGGGGCGCTCATCGCTCGGCGGGGCGCAGGGCCGGGCAGCGGAGGGCGGGGGCTGGC
GGCGCGGCAGCAGGAAAGTTTTGCAGCCGCTGAGCCAGGAGCTTCTTTCTCCGAGAGCCCAGAC
GGAAAATAAAAACTTTCCGGAAAAGTCCCTGGGCTGGGGGAGGGCAAGGGGATGAGGATCGGGA
GGGGAGGGGACTAAGGGGAGGAGATGGGGAGGAGGGAAAATGGCCGAGGCGGGAGGACTCTGAG
GCCCGGAACGCGGCAGCCGGCTCGGGGCCGCCGGCACTGGGGAGGCGCCGCCGCCGCCAAGAAT
CTGCCGCCTGGCGCGCTCGGAAGGACGGGGCGACGGGTCGGCGCGGCCCGAGCGCCGGGCGGGG
GCCGGGGCGGGAAGGGGGCGTGAGGGGCGGGGAAGGGGAGCTGGGGGCGGGCACTGGGGAGCCA
CCACCCGGCGGGCGCGGGAGTCGCAGGAGCAGCGGCCGCAGTGGCCGCAGGGTGCCCGGTCGCC
GCCCCCTCCGCCCGCAGTTCCCGGGGCCTGAGCCTCCGCGAGCCGGGATAGGACCGAGTGGCGG
GGCTCGGAGCCGCCTCGGCAGGCGCGGCCCTTCCCTCCCTGCAGGGATTCCCGTCCTCTCGGGT
CCCGCTGCCCCGCCCCGACTGCGGACGGAGTGGATGGGGTGCACCGCCTCAGACCCGCTTCCTG
GGGTCGCGAACTTCCCGGGCCCCAAGGTCCCTGCCACCTCTTCCAAATGCTGGAGGACTACGCA
CTTCTAC
```

Figure S2, relates to Figure 4: **Mapping of the NF-κB site in the *PRKD1* promoter region -1 to -1030. A:** Control blots (input control) for Fig. 4A. Cell lysates were analyzed by Western blot for overexpression of p65 (anti-p65), as well as for β-actin (anti-β-actin; loading control). **B:** Nucleotide sequence of the *PRKD1* promoter (-1 to -1030) fused to a luciferase reporter. Blue labeling indicates the fragment identified in Fig. 4A as relevant for p65-mediated activation of the *PRKD1* promoter. The potential reverse oriented NF-κB binding site within this fragment is indicated in red.
